# Clinical and radiological results of high tibial of osteotomy over the age of 65 are comparable to that of under 55 at minimum 2-year follow-up: a propensity score matched analysis

**DOI:** 10.1186/s43019-024-00214-9

**Published:** 2024-02-28

**Authors:** Jae-Young Park, Jae-Hwa Kim, Jin-woo Cho, Min soo Kim, Wonchul Choi

**Affiliations:** Department of Orthopaedic Surgery, CHA University, CHA Bundang Medical Center, 351 Yatap-Dong, Bundang-gu, Seongnam-si, Gyeonggi-do Republic of Korea

**Keywords:** High tibial osteotomy, Age, Osteoarthritis

## Abstract

**Purpose:**

The results of medial open-wedge high tibial osteotomy (MOWHTO) according to age is inconclusive. This study aimed to compare the clinical outcomes and failure of MOWHTO in patients < 55 years and > 65 years.

**Methods:**

Consecutive patients who underwent MOWHTO from July 2009 to August 2020 were retrospectively analyzed. 205 patients were considered for analysis. A 1-to-1 propensity score matched analysis to assess clinical outcomes scores including International Knee Documentation Committee (IKDC) subjective score and Lysholm score, radiologic outcomes, complication, and Total Knee Arthroplasty (TKA) conversion between patients > 65 years and patients < 55 years was performed. Radiologic outcomes included Hip-Knee-Ankle (HKA) angle, Weight Bearing Line ratio (WBLR), posterior tibial slope (PTS), and Insall-Salvati (IS) ratio before and after surgery.

**Results:**

The follow-up period was 50.4 months in patients > 65 years and 55.3 months in patients < 55 years. There was no significant difference in the preoperative and postoperative HKA angle, WBLR, PTS, IS ratio, IKDC score and Lysholm score between the two groups. The arthroscopic evaluation of cartilage did not show any statistically significant differences between the two groups. Regarding Minimal clinically important differences (MCID), in the 26% of the older group exceeded MCID of IKDC score; 45% of the older group exceeded MCID of Lysholm score. In the younger group, 24% exceeded MCID of IKDC score and 35% exceeded MCID of Lysholm score. In older group, there were 7 (11.3%) cases of TKA conversion while no TKA conversion was recorded in the younger group. (*P* = 0.007) The average time to TKA conversion was 67 months. (42 months to 90 months) Kaplan–Meier analysis revealed that the survival rate was 95.2% at 4 years in the older group.

**Conclusion:**

Similar clinical results were obtained in patients over 65 years of age that were eligible for MOWHTO at minimum 2-year follow-up as in patients under 55 years of age. MOWHTO may be a viable option in older patients if proper indications are met. However, the risk of TKA conversion must be considered preoperatively and discussed with patients.

*Study Design*: Cohort study; Level of evidence, 3.

## Introduction

High Tibial Osteotomy (HTO) is a representative surgical treatment for medial compartment osteoarthritis. It has been known to be particularly suitable for relatively young middle-aged patients where the aim is to postpone the need for initial joint replacement surgery in order to decrease the likelihood of further revision surgeries in the future [1–4]. The ideal age range for medial open wedge HTO (MOWHTO) is known to be 40 to 60 years [5]. However, with the aging population and the desire to preserve the natural knee joint, MOWHTO has been recommended for patients over the age of 60 as well [6–8].

Some authors emphasized a significantly higher failure rate in patient over 65 years, and certain study showed the risk of HTO's failure rate increasement by 7.6% per each year of age [9–11]. Thus, some studies advocate an upper age limitation for HTO to be 65 years [12, 13].

There are increasing evidence that age does not affect clinical or radiological outcomes after HTO [7, 8, 14]. Goshima et al. advocated there were no statistical differences in clinical outcomes between patients that were older than 65 and less than 65 [7]. Similarly, Khon et al. reported that there were no statistical differences between average 57 years old group and the 42 years old group in clinical outcomes after HTO [14].

Little evidence is present that have compared the post-operative clinical outcomes of HTO in different age groups using patient-matched data. This makes it uncertain whether HTO is a suitable procedure for older patients. To address this, a propensity score matching (PSM) based on pre-operative characteristics would be useful in comparing post-operative clinical outcomes between age groups. The variables used in the matching process should include gender, body mass index (BMI), and osteoarthritis (OA) grade, as these have been identified as potential confounding factors that can significantly impact post-operative clinical scores in HTO.

This study aimed to evaluate the clinical and radiological outcomes after MOWHTO in different age groups. PSM was used for comparing the post-operative patient reported clinical outcomes scores between different age groups. It was hypothesized that the clinical score after 2 years of MOWHTO in younger age group (under 55 years) would be comparable to that of older age group. (over 65 years).

## Methods

**Fig. 1:**
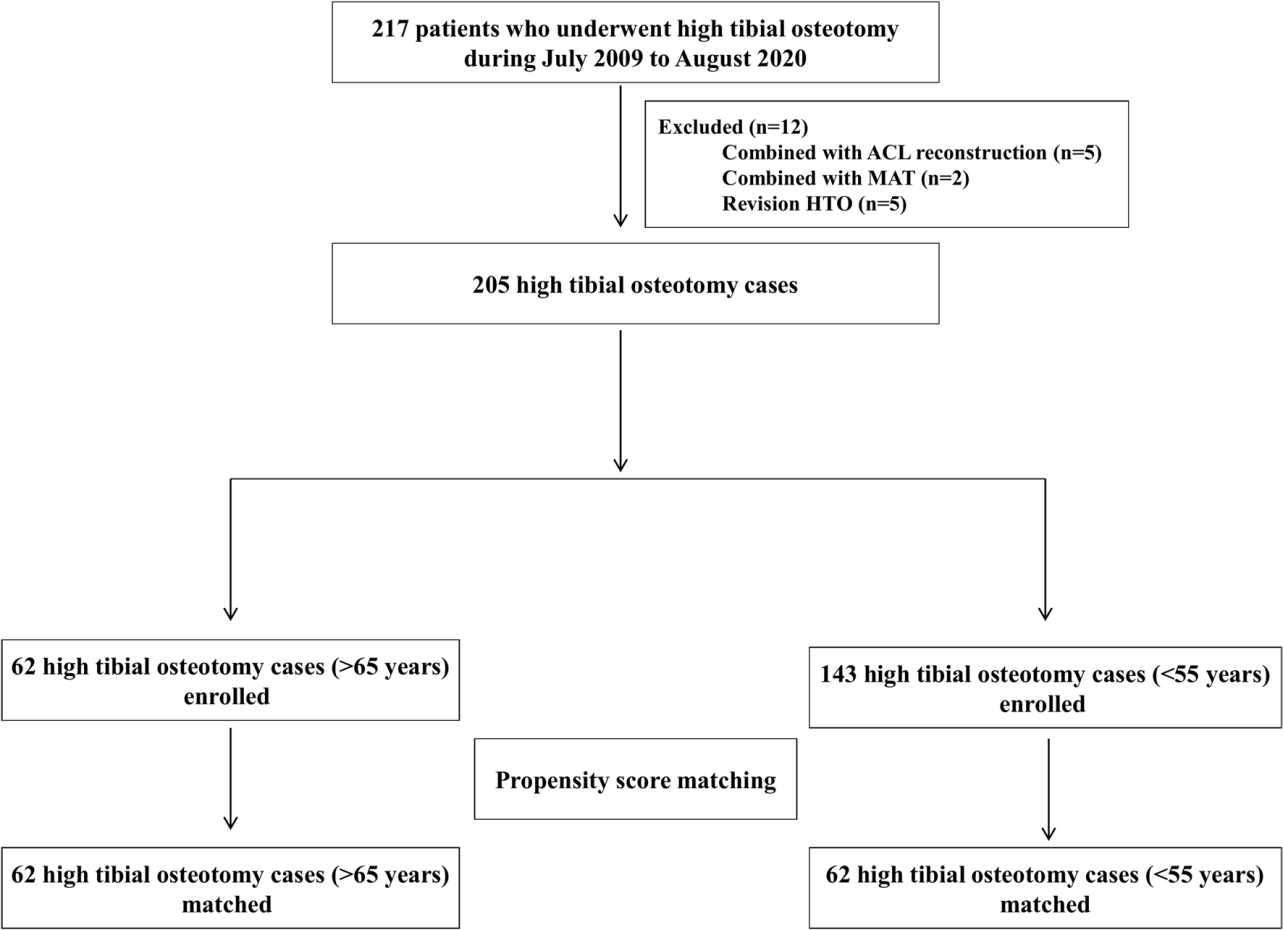
Flowchart of enrollment. *ACL* anterior cruciate ligament, *MAT* meniscus allograft transplantation, *HTO* high tibial osteotomy

After obtaining Institutional Review Board approval, the data was obtained from the––medical center orthopedic department, between July 2009 and August 2020. This study retrospectively reviewed data of enrolled patients who met the following inclusion criteria: (1) underwent MOWHTO with 2nd look arthroscopy due to isolated medial compartment knee osteoarthritis, (2) with preoperatively varus limb malalignment, (3) BMI under 30(kg/m^2^), (4) and have active compliance with over 2-Year postoperative follow up with clinical and radiographic data. 5 cases combined with anterior cruciate ligament reconstruction, 2 case combined with medial meniscal allograft transplantation, 5 cases of revision cases (Fig. 1).

### Propensity score matching and characteristics of patients

In this study, patients who met the criteria during the index period was total 205 in our institution. These patients were divided into two groups according to the ages. PSM was performed to minimize selection bias for MOWHTO between two groups. It included patient factors that may have affected the outcome of the surgery, and the matching was performed based on the calculated propensity score. The propensity score was estimated by running a logistic regression model. Before PSM, there were 62 patients of older group (> 65 years) and 143 patients of younger group (< 55 years) respectively. Older age was set to be greater than 65 because previous studies have reported inferior outcomes after HTO in age > 65. Younger age was set to be less than 55 because studies have reported superior outcomes after HTO in patients in their 50 s [15–17]. The mean BMI were 25.6 ± 2.6 and 26.4. ± 3.6 (kg/m^2^), and the mean per-operative Hip-Knee-Ankle (HKA) varus angle degree was 7.19 ± 3.3 and 5.6 ± 3.5 retrospectively. There were significant differences in preoperative HKA angle (*p* < 0.001) before PSM matching. (Table 1) However, there were no significant differences for each possible confounder including the patient-related factors that of age, gender, BMI, Kellgren Lawrence (KL) grade, and prevalence of hypertension (HTN) and diabetes mellitus (DM) between the two groups with unmatched propensity scores. Consequently, after all variables were successfully matched, 62 patients in each group were enrolled for the PSM analysis (by 1-to-1 matching). Men and women were matched with 16 and 46 respectively, and HTN patients were 23 in older group and 22 in younger group. DM prevalence was the same, six patients each. The student's t-test and chi-square test were used to analyze the difference of these factors, and there was no statistical difference between older group and younger group.

**Table 1:** Summary of data before and after propensity score matching

	Before propensity score matching		P-value	Propensity Score-Matched (1:1)		*P*-value
	Older group (> 65 yrs, *N* = 62)	Younger group (< 55 yrs, *N* = 143)		Older group (> 65 yrs, *N* = 62)	Younger group (< 55 yrs, *N* = 62)	
Sex(Male/Female)	16/46	55/88	0.12	16/46	16/46	1
BMI (Mean ± SD)	25.6 ± 2.6	26.4 ± 3.6	0.07	25.6 ± 2.6	25.5 ± 3.2	0.82
Preop HKA (degree)	7.2 ± 3.3	5.6 ± 3.5	< 0.001	7.2 ± 3.3	6.6 ± 3.3	0.67

### Surgical procedure and post-operative rehabilitation

The operation was performed by two different senior surgeons. With supine position and limb tourniquet during surgery, prior to MOWHTO, diagnostic arthroscopy was conducted before osteotomy to evaluate the extent of cartilage degradation in the medial, lateral, and Patellofemoral compartments, followed by microfracture or meniscectomy, if needed. Osteotomy procedure was performed through the open-wedge technique using the antero-medial approach under the C-arm guidance. The osteotomy was started 35–40 mm below the medial articular surface of the tibia. A transverse osteotomy was performed from the medial cortex to the upper third of the proximal tibiofibular joint using the ascending biplanar technique. The gap was gradually opened aiming for a post-operative weight-bearing axis fixed at a point lateral spine of the tibial plateau and then fixed with TomoFix device (DePuy Synthes) An active & passive knee range of motion and active quadriceps strengthening exercise started the day after surgery. Patients were placed in a hinged knee brace postoperatively, tolerable partial weight-bearing with crutches until 4 weeks. Full weight-bearing permitted after 4 weeks of surgery.

### Radiographic assessment

Radiographic study was performed by 2 observers. The HKA angle, Weight Bearing Line ratio (WBLR), posterior tibial slope (PTS), and Insall-Salvati (I-S) ratio before and after surgery was measured and compared between two groups. These measurements were performed as follows: The HKA angle is measured by the angle between the mechanical axis of femur and tibia. The WBLR is calculated by measuring the distance from the medial side of the tibia plateau to the point of the weight-bearing line intersects the proximal tibia, and dividing the measurement by the entire width of the tibia plateau. PTS was measured by utilize for standard lateral knee radiographs. The line interconnecting the central point in the bone marrow cavity at the boundary of the metaphyseal-diaphyseal of the proximal tibia and that of distal tibia, was considered as the lateral anatomical axis of the tibia, and the PTS was measured using tibia plateau and the tibia lateral anatomical axis. The I-S ratio is the ratio of the length of the patellar tendon (measured from tibial tuberosity to the proximal patellar bone) to the maximum length of the patellar bone (measured from distal to proximal length of patella bone).

### Arthroscopic cartilage evaluation

The preoperative condition of the cartilage was confirmed through an arthroscopic examination performed just before the MOWHTO, followed by meniscectomy or microfracture, if needed. And the postoperative condition of the cartilage was confirmed through an arthroscopic examination performed with the plate removal surgery. Plate removal with diagnostic arthroscopy were done at 13.1 months and 13.6 months after surgery in old age and young age group, respectively(*P* = 0.271), and the cartilage status were recorded in each compartment according to the International Cartilage Repair Society (ICRS) classification for each compartment [18]. (medial and patellofemoral) In addition, the degree of change in the ICRS grade was recorded according to change of cartilage in each compartment into three categories: improved, unchanged, and worsen.

### Clinical evaluation

Clinical evaluation was evaluated using Lysholm scores and International Knee Documentation Committee (IKDC) subjective score before and at 24 months after surgery. The preoperative evaluation was conducted through physical examination and a direct questionnaire, and the scores after 24 months of evaluation was performed through standardized questionnaires by outpatient care. In addition, radiographs, laboratory tests, and medical records from the first 6 weeks after surgery were reviewed to identify wound problems (delayed wound healing, cellulitis), surgical complications (hinge fracture. plateau fracture, neurovascular injury, intra-articular screw penetration). Data for at least 12 months after surgery were reviewed to identify nonunion, hardware failure, and infection that occurred within the period. Conversion to TKA was also reviewed. Conversion to TKA was considered in patients with inadequate relief of pain with progressive joint damage.

### Statistical analysis

Kaplan–Meier survivorship curves with log-rank tests were constructed to estimate survivorship. A propensity score matching was performed by SAS (version 9.2, SAS Institute, Cary, North Carolina). Other statistical analyses were conducted with IBM SPSS software package (version 27.0). As for continuous variables, the Kolmogorov–Smirnov test was first applied to test normality. The independent t test was used to statistical comparisons of a difference existed in Age, BMI, HKA, WBLR, PTS, I-S ratio, follow up periods, a duration until 2nd look arthroscopy, IKDC and Lysholm score between two groups. To confirm dependence between variables, the Pearson chi-square test was used to analyze the demographic data between groups; include sex, HTN, DM, conversation to Total Knee Arthroplasty (TKA), complications. a duration until 2nd look arthroscopy. The Mann–Whitney U test was used to compare differences in ordinal scales that of K-L and ICRS grade. Minimal clinically important difference (MCID) for the IKDC score (Δ12.5) and Lysholm score (Δ8.9) were used to assess clinical relevance and significance [19].

## Results

The study population consisted of 62 participants in each of two age groups: an older group (> 65 years) and a younger group (< 55 years). The follow-up period was 50.4 months in the older group and 55.3 months in the younger group. (Table 2) The mean age of the older group was 68.9 ± 3.3 years, which was significantly higher than that of the younger group (51.7 ± 2.9 years, p < 0.001). There was no significant difference between the two groups in terms of sex, BMI, hypertension, or diabetes mellitus. The K-L grade distribution also did not differ significantly between the two groups. Furthermore, there were no significant differences between the two groups in the incidence of concomitant partial meniscectomy or concomitant microfracture.

**Table 2:** Demographics of study population

	Older group (> 65 yrs, *N* = 62)	Younger group (< 55 yrs, *N* = 62)	*P*-value
Age	68.9 ± 3.3	51.7 ± 2.9	< 0.001*
Sex(Male/Female)	16/46	16/46	n.s
BMI	25.6 ± 2.6	25.5 ± 3.2	n.s
HTN	23	22	n.s
DM	6	6	n.s
K-L grade (2/3/4)	29/32/1	28/26/8	n.s
Concomitant Partial meniscectomy (MM)	14	16	n.s
Concomitant Microfracture	11	8	n.s
Follow period (months)	50.4 ± 26.1	55.3 ± 27.4	n.s

The values are means ± standard deviations

*BMI* body mass index, *HTN* hypertension, *DM* diabetes mellitus, *K-L* Kellgren Lawrence, *MM* medial meniscus, *n.s* not significant

Concerning radiographic outcomes, the two age groups were compared and the results are presented in Table 3. There was no significant difference in the preoperative and postoperative HKA angle, preoperative and postoperative WBLR, preoperative and postoperative PTS, and preoperative and postoperative I-S between the two groups. The arthroscopic evaluation of cartilage using the ICRS grading system did not show any statistically significant differences between the two age groups. (Table 3) There was no significant difference in the preoperative and postoperative IKDC score and Lysholm score. (Table 4).

**Table 3:** Radiographic and ICRS outcomes of the two groups

	Older group (> 65 yrs, *N* = 62)	Younger group (< 55 yrs, *N* = 62)	*P*-value
Preop HKA(degree): Varus	7.2 ± 3.3	6.6 ± 3.3	n.s
Postop HKA(degree): Valgus	2.0 ± 4.0	3.1 ± 3.4	n.s
Preop WBLR(%)	22.2 ± 15.7	23.2 ± 12.6	n.s
Postop WBLR(%)	55.9 ± 16.8	59.3 ± 17.4	n.s
Preop PTS(degree)	12.1 ± 3.6	10.2 ± 4.4	n.s
Postop PTS(degree)	11.1 ± 4.1	9.7 ± 4.5	n.s
Preop I-S(ratio)	1.0 ± 0.2	1.0 ± 0.2	n.s
Postop I-S(ratio)	1.1 ± 0.2	1.1 ± 1.0	n.s
Preop ICRS(MFC)	0/6/13/16/27	0/9/22/20/11	n.s
Postop ICRS of MFC	0/9/22/20/9	0/7/31/14/10	n.s
Delta ICRS(Improved/Unchanged/Worsen): MFC	21/36/5	27/30/4	n.s
Preop ICRS of PF	14/24/6/11/7	21/17/13/7/4	n.s
Postop ICRS of PF	14/28/8/8/4	19/21/12/8/2	n.s
Delta ICRS(Improved/Unchanged/Worsen): PF	3/55/4	5/50/6	n.s

The values are means ± standard deviations

*HKA* Hip-Knee-Ankle, *WBLR* Weight Bearing Line ratio, *PTS* posterior tibial slope, *I-S* Insall-Salvati, *ICRS* International Cartilage Repair Society, *MFC* medial femoral condyle, *PF* patellofemoral, *n.s* not significant

**Table 4:** Clinical outcomes of the two groups

	Older group (> 65 yrs, *N* = 62)	Younger group (< 55 yrs, *N* = 62)	*P-value*
Preop IKDC	37.2 ± 16.7	40.2 ± 18.4	n.s
Postop IKDC 2 year	51.5 ± 19.8	54.9 ± 15.1	n.s
Delta IKDC	13.2 ± 19.5	14.0 ± 12.4	n.s
Preop Lysholm	43.9 ± 23.2	48.7 ± 21.3	n.s
Postop Lysholm 2 year	62.1 ± 22.5	62.4 ± 19.1	n.s
Delta Lysholm	17.9 ± 29.0	13.8 ± 18.6	n.s

The values are means ± standard deviations

*IKDC* International Knee Documentation Committee, *n.s* not significant

Regarding clinically relevant values of the MCID, in the older group 16 of 62 patients (26%) showed a IKDC score exceeding 12.5; 28 of 62 patients (45%) revealed a Lysholm score exceeding 8.9. In the younger group, 15 of 62 patients (24%) showed a IKDC score exceeding 12.5; 22 of 62 patients (35%) revealed a Lysholm score exceeding 8.9. There were no significant differences in the two groups in the percentage of patients that exceeded MCID for both IKDC and Lysholm scores.

There was no significant difference in the incidence of lateral hinge fractures between the two groups (18 in the older group vs 15 in the younger group, p > 0.05). Additionally, there were no cases of delayed union or nonunion in either group. The incidence of wound problems was low, with only 1 case reported in the older group and none in the younger group. No cases of infection were reported in either group.

**Fig. 2:**
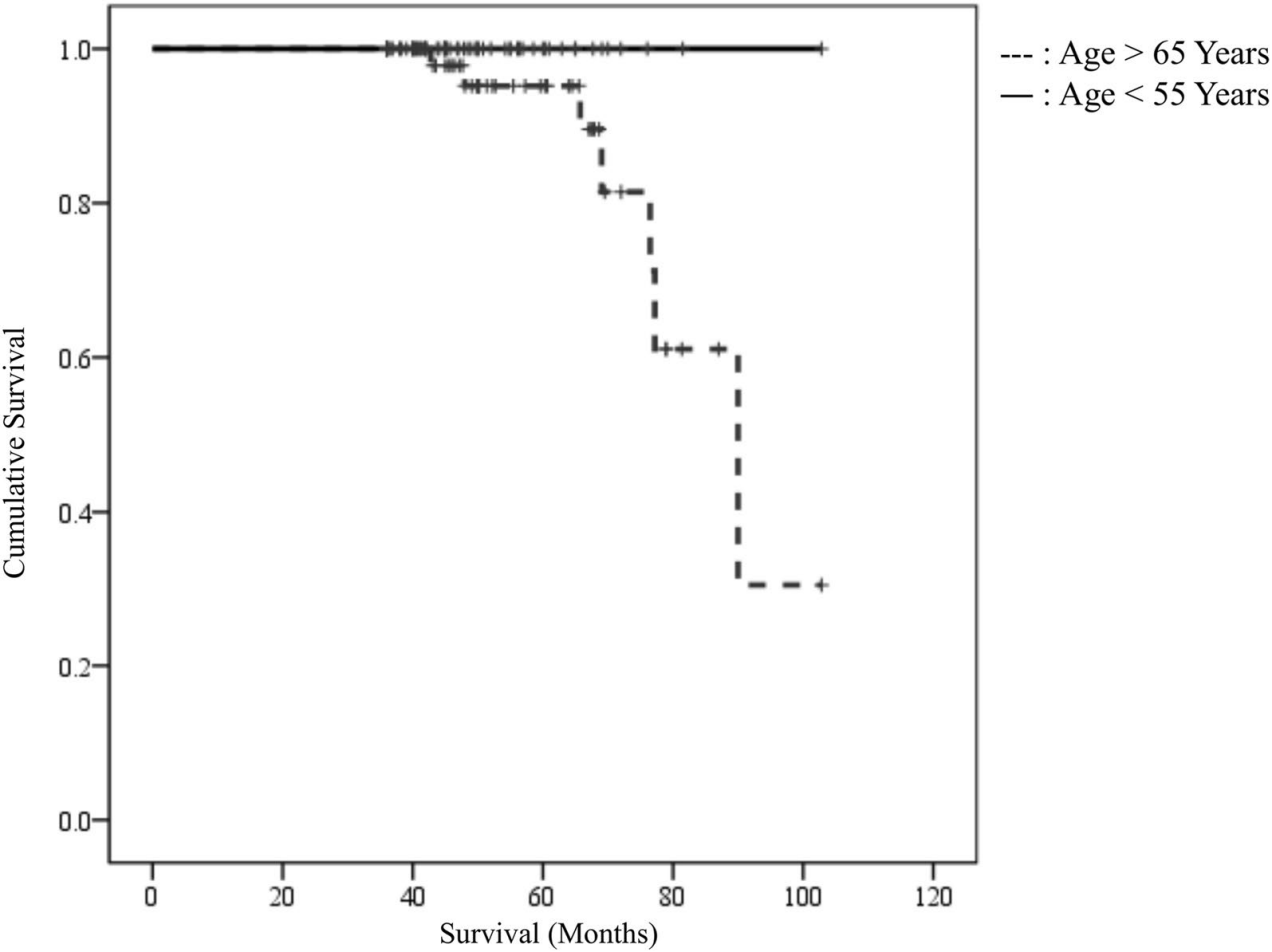
Kaplan–Meier survival analysis for open wedge high tibial osteotomy in 65 > years and 55 < years

In older group, there were 7 (11.3%) cases of TKA conversion while no TKA conversion was recorded in the younger group. (P = 0.007) The average time to TKA conversion was 67 months. (42 months to 90 months) Kaplan–Meier analysis revealed that the survival rate was 95.2% at 4 years in the older group. (Fig. 2).

## Discussion

The most important finding of this study is that the radiological outcome and clinical outcomes of MOWHTO were comparable between the age group over 65 years and the age group below 55 years.

The purpose of this study was to evaluate whether age has a major influence on the clinical outcomes of MOWHTO. Clinical, radiological, and arthroscopic findings of MOWHTO performed on matching groups paired with older and younger patients were compared. The main result of this study was that similar levels of improvement were shown after the same period in MOWHTO postoperative IKDC and Lysholm scores in old age and young age after PSM for gender, BMI, and HKA angle. Thus, the original hypothesis has been invalidated, and in mid-term follow-up, MOWHTO may be a feasible treatment under appropriate surgical targets and indications for medial compartment OA in patients over 65 years of age.

Goshima et al. noted age as an important factor in the clinical prognosis of MOWHTO. Sixty of consecutive MOWHTOs were performed using TomoFix plate and clinical and radiographic results were compared for 26 knees in 23 patients over 65 (mean age of 68.7 ± 2.9 years at surgery) and 34 knees in 27 patients under 65 (mean ago of 56.2 ± 7.5 years at surgery). The use of the Japan Orthopedic Association's knee score and Oxford knee score as comparative indicators showed that both scores improved in all groups after surgery, and age was independent of prognosis and radiologic outcomes [7]. Kohn et al. also argued that age was not necessary as an indication for HTO in their retrospective study [14]. Song et al. also reported that cartilage status, rather than chronologic age determined the outcomes of MOWHTO [8].

In the present study, although the clinical and radiological outcomes were similar between the two groups, conversion to TKA was seen only in the older age group. This result was in line with many previous studies. A study by Keenan et al. also noted that age older than 47 was an independent predictor of failure in MOWHTO [12]. A study by Hui et al. also stated that age less than 50 years was an independent factor associated with long-term survival of HTO [9]. A Finnish registry-based study also reported that patients aged > 50 years had worse survivorship after HTO [10]. Many previous studies indicate older age itself as a risk factor for TKA conversion after HTO [20, 21]. However, when contemplating that the clinical outcome results are similar between the two groups, the difference in conversion to TKA may be due to the reimbursement criteria of the national health insurance system which allows patients over 60 years to undergo TKA [22]. The results of the present study and the results of previous studies show that when other factors are accounted for, the mid-term results are similar between younger are older age groups [1].

Given the similar clinical results but inferior survivorship in the older group, the decision to continue non-operative treatment and consider TKA later if the conservative treatments fail or perform HTO in patients’ groups over 65 years old should be contemplated by the surgeon and discussed with the patient. It should be an interesting area for further research.

While there is increasing evidence that age does not affect clinical or radiological outcomes after HTO, still high-quality evidence is lacking. This study aimed to evaluate the clinical and radiological outcomes after MOWHTO in different age groups by using a PSM method. What can be insisted by the present study is that when OWHTO was performed in the patient group over 65 years old, the short-term follow-up surgical results at 2 years were similar to those of the patient group under 55 years old, and the TKA conversion rate was 11% at mean f/u of 50 months. Long-term follow-up results are unknown and additional research will be needed.

There are some limitations to the present study. First this was a retrospective study with the risk of selection bias. Although a PSM analysis was performed to avoid any bias, a prospective is still needed to confirm a definitive conclusion. Second, the number of patients was relatively small. Third, two surgeons performed the surgeries. This may have affected as a bias. Fourth, the follow-up duration varied and was broad. Fifth, the basis for determining the groups as 55 and 65 years old was arbitrary and could have acted as a bias.

## Conclusion

Similar clinical results were obtained in patients over 65 years of age that were eligible for MOWHTO at minimum 2- year follow-up as in patients under 55 years of age. MOWHTO may be a viable option in older patients if proper indications are met. However, the risk of TKA conversion must be considered preoperatively and discussed with patients.

## Data Availability

The data that support the findings of this study are available upon reasonable request.
